# Psychiatric admission decrease during COVID-19 lockdown in older patients

**DOI:** 10.1192/j.eurpsy.2023.538

**Published:** 2023-07-19

**Authors:** O. Martin-Santiago, M. C. Vallecillo-Adame, T. Jimenez-Aparicio, A. Perez-Escudero

**Affiliations:** ^1^Department of Psychiatry, Hospital Clínico Universitario de Valladolid, Valladolid; ^2^Complejo asistencial de Zamora, Zamora, Spain

## Abstract

**Introduction:**

Coronavirus disease 2019 (COVID-19) modify voluntary admission rates to psychiatric wards in the early phases following pandemic onset. Older patients have higher COVID-19 distress scores because elderly people are at risk for COVID-19 infection.

**Objectives:**

The present investigation aimed at admission rates of elderly patients to a General Hospital Psychiatric Ward during the lockdown due to the COVID-19, compared to similar periods of 2018 and 2019.

**Methods:**

Anonymized data on psychiatric admissions (n=55) from one general hospital psychiatric ward have been obtained and analysed. We compared admission characteristics between April and June of 2018 and 2019 with the same period of 2020 (lockdown).

**Results:**

During the COVID-19 lockdown, a significant reduction in psychiatric hospitalizations of older patients (aged >65 years) was observed in the lockdown (69.2%; χ2=4.823,df=1,p=0.028) in contrast with young patients (26.7% reduction). There was a reduction of 14% in admission due to suicidal behaviour (IRR = 0.57; 95% CI: 0.11-2.75; p=0.48), 25% in depression (IRR = 0.28; 95% CI: 0.64-1.25; p=0.09) and 50% in psychotic disorders (IRR = 0.33; 95% CI: 0.07-1.48; p=0.15). There was none admission by dementia during the lockdown.

**Conclusions:**

Changes in the number of psychiatric admissions, particularly for older patients, were observed during the COVID-19 lockdown. During this period, their fear levels could modify their psychiatric admission rates. We suggest that the decrease of psychiatric admissions in the elderly was due to fear of contagion in hospitals.

**Disclosure of Interest:**

None Declared

